# A cross-sectional survey on sleep quality, mental health, and academic performance among medical students in Saudi Arabia

**DOI:** 10.1186/s13104-019-4713-2

**Published:** 2019-10-21

**Authors:** Abdullah Murhaf Al-Khani, Muhammad Ishaque Sarhandi, Mohamed Saddik Zaghloul, Mohammed Ewid, Nazmus Saquib

**Affiliations:** 1 College of Medicine, Sulaiman AlRajhi Colleges, P.O. Box 777, 51941, Al Bukayriah, Saudi Arabia; 2 Internal Medicine Department, Faculty of Medicine, Internal Medicine Hospital, Cairo University, Kasr A. Ainy St., Cairo, 11562 Egypt

**Keywords:** Academic performance, Medical students, Mental health, Sleep quality

## Abstract

**Objective:**

Adequate sleep is integral to better mental health and facilitates students’ learning. We aimed to assess sleep quality among medical students and to see whether it was associated with their mental health (e.g., depression, anxiety, and stress) and academic performance.

**Results:**

A total of 206 responded, and 95 of them had complete data on demography, lifestyle, academic performance, sleep quality (Pittsburgh Sleep Quality Index), and mental health (Depression Anxiety Stress Scales). The prevalence of poor sleep was 63.2%; it was higher among students who were physically inactive and had more screen time. Poor sleepers demonstrated higher academic performance than sufficient sleepers (*p* = 0.04). The prevalence of depression, anxiety, and stress were 42%, 53%, and 31% respectively. Sleep quality was significantly associated with depression (*p* = 0. 03), anxiety (*p* = 0.007), and stress (*p* = 0.01).

## Introduction

Sleep is central to health and well-being [1]. For older adults (46–60 years), the recommended sleep duration is 7 h or more per night on a regular basis; for younger adults (18–45 years), an even longer duration (> 9) is considered appropriate [2]. Sleep greatly influences mental function, and thus, affects performance. Insufficient sleep decreases general alertness, impairs attention, and slows cognitive processing [3, 4].

All available Saudi Arabian studies on sleep quality among medical students were conducted at governmental colleges; these studies used disparate instruments for the assessment of sleep quality [5–9]. For example, studies that used the Pittsburgh Sleep Quality Index (PSQI) reported that three-quarters (70–76%) of medical students were poor sleepers [7–9].

Both sleep quality and academic performance can affect students’ mental wellbeing [10, 11]. In private medical colleges, where the students are mostly non-Saudi, there are additional challenges to mental health, such as stress about tuition fees. Scholarships to cover tuition fees are available, but students must obtain and/or maintain academic excellence to qualify. This fact likely adds mental pressure on students above and beyond what they feel by being in the program itself.

Therefore, in this paper, we assessed sleep quality, academic performance, and mental health in a sample of medical students enrolled in a private medical college in the Al-Qassim region of Saudi Arabia.

## Main text

### Methods

#### Design, ethical consideration, and data collection

This was an online cross-sectional survey administered to the students enrolled in the medical program at Sulaiman AlRajhi Colleges, Al-Qassim, Saudi Arabia. The college research committee reviewed and approved the study protocol (SRC/01/2018/008). The study was conducted in March of 2018.

The survey was sent to all enrolled medical students (n = 446) via their college e-mail. The students could access the survey with any electronic device that was capable of accessing their email. The survey began with a brief description of the study itself, followed by a detailed informed consent that explained the participants’ rights, the perceived risks and benefits of participation, and the measures taken by the investigators to keep their personal information confidential. The students could access the survey only after they agreed to this informed consent. It took a participant around 10 min to fill out the survey.

Participants had to be at least 18 years old (inclusion criterion) and currently enrolled in the medical program. Students with a chronic medical condition were not eligible (exclusion criterion). A chronic medical condition was defined as a self-reported presence of any of the following conditions: diabetes mellitus, hypertension, or bronchial asthma.

A total of 206 students responded to the survey (rate 46%); nine did not meet the eligibility criteria (one student was < 18 years old and another eight reported chronic medical illnesses). A total of 102 were excluded for missing data (missing data on all variables = 67; missing data on key variables such as sleep, mental health, and academic performance related questions = 35). Therefore, 95 students who had complete data on all variables were included in the analyses.

#### Questionnaire contents

The survey included, among other questions, two validated and widely used screening tools: (1) the Pittsburgh Sleep Quality Index (PSQI) [12], and (2) the Depression Anxiety Stress Scales (DASS-21) [13]. The PSQI is a tool used to evaluate two sleep domains: sleep quality and sleep disturbances. The questionnaire yields an overall score ranging from 0 to 21. A score of 5 or greater was indicative of poor overall sleep quality, whereas a score of less than 5 was indicative of good overall sleep quality.

The DASS-21 is a clinical assessment tool that measures psychological distress. It contains 21 items divided into three subscales of depression, anxiety, and stress, with 7 items allocated for each subscale. The items are scored on a 4-point scale ranging from 0 (did not apply to me at all) to 3 (applied to me very much, or most of the time). The range of score a participant could get for each subscale varied from 0 to 21. The recommended cutoff points were used to classify participants into normal, mild, moderate, severe, and extremely severe in terms of depression, anxiety, and stress [14].

Academic performance was assessed with a self-reported grade point average (GPA) in the last year. GPA is a well-established method to assess academic performance in research [11, 15, 16]. At SRC, a minimum GPA of 4.0 is required to maintain a college scholarship. Therefore, it was used to categorise the students into high (GPA ≥ 4.0) and low (GPA < 4.0) achievers. Additionally, students were asked about a number of socio-demographic variables seen in Table 1.

**Table 1:** Comparison of demographic, lifestyle and academic characteristics between good and poor sleepers according to the Pittsburgh Sleep Quality Index scale (n = 95)

	Sleep quality		*p* value
	Good sleepers (n = 35), %	Poor sleepers (n = 60), %	
Gender			
Male	82.9	71.7	0.219
Female	17.1	28.3	
Academic year			
PYP	14.3	23.3	0.465
Preclinical years	60.0	48.3	
Clinical years	25.7	28.3	
Dietary habits			
Unhealthy	51.4	65.0	0.193
Healthy	48.6	35.0	
Physical activity			
Inactive	22.9	48.3	*0.014*
Active	77.1	51.7	
Smoking			
No	85.7	90.0	0.529
Yes	14.3	10.0	
Screen time			
Up to 5 h	40.0	26.7	*0.046*
Five to 10 h	51.4	43.3	
More than 10 h	8.6	30.0	
Quran reading			
No	11.4	21.7	0.209
Yes	88.6	78.3	
Living condition			
Alone	8.6	16.7	0.528
Roommate	71.4	63.3	
Family	20.0	20.0	
Attendance			
Irregular	5.7	11.7	0.339
Regular	94.3	88.3	
Teamwork			
All the time	11.4	8.3	0.525
Sometimes	85.7	83.3	
Not at all	2.9	8.3	
GPA			
High achievers	37.1	58.3	*0.046*
Low achievers	62.9	41.7	

#### Data analysis

The statistical analysis for our study was carried out using SPSS software version 25 for Windows. The significance level was set at 5%. The characteristics of the sample were summarised using means and standard deviations (SD) for continuous variables and frequencies with percentages for categorical variables. Independent sample *t* test was used for continuous variables while Chi square test (χ^2^) was used for categorical variables. Univariate and multivariate regression analyses were used to model the predictors of academic performance.

### Results

#### Socio-demographic characteristics

Male students constituted around 76% of the sample. The sample’s mean age was 20.8 years (standard deviation = 1.95, range = 18–28 years). The preparatory, preclinical (years 1–2), and clinical (years 3–5) students made up 20.0%, 52.6%, and 27.4% of the sample, respectively.

Thirty-nine percent of the students described their lifestyle as either ‘very’ or ‘somewhat’ inactive; 60% described their dietary habits as ‘unhealthy.’ Smoking was reported by 11.6% of the students. Less than half (46.3%) reported a screen time of 5 to 10 h/day. Approximately two-thirds of the students (66%) were sharing a room with a roommate, 20% lived with their families, and 14% lived alone. A summary of demographic and lifestyle characteristics is shown in Table 1.

#### Academic performance

The overwhelming majority of the students reported regular attendance at academic activities (90.5%) and reported enjoying working in group academic activities (93.7%). The sample had almost equal proportions of high (50.5%) and low (49.5%) achievers.

#### Subjective sleep quality (PSQI)

The PSQI score ranged from 0 to 16 (mean = 5.7, SD = 3.2); 36.8% were good sleepers. Sleep quality was significantly associated with physical activity level (*p* = 0.014) and screen time (*p* = 0.046). Gender, academic year, dietary habits, smoking status, Quran reading, and living condition did not differ significantly between poor and good sleepers. Sleep quality was significantly associated with reported GPA (*p* = 0.046). Significance values of subjective sleep quality compared with sample demographic/lifestyle characteristics and academic performance are presented in Table 1.

#### Depression Anxiety Stress Scales (DASS-21)

The participants’ depression scores ranged from 0 to 20 (mean = 5.31, SD = 4.89). 27.3% of the participants had mild to moderate depression and 14.7% had severe to extremely severe depression. The anxiety scores ranged from 0 and 19 (mean = 4.86, SD = 4.27). Only 3% had mild to moderate anxiety and 25.3% had severe to extremely severe anxiety. Lastly, the stress scores ranged between 0 and 21 (mean = 6.18, SD = 4.86); 17.9% had mild to moderate stress and 12.7% had severe to extremely severe stress. Sleep quality was significantly associated with depression, anxiety, and stress (*p* = 0.013), (*p* = 0.003), (*p* = 0.004), respectively. Table 2 shows significance values of subjective sleep quality compared with DASS-21.

**Table 2:** Comparison of mental health status (depression, anxiety, and stress) across sleep quality (n = 95)

	Sleep quality		*p* value
	Good sleepers (n = 35), %	Poor sleepers (n = 60), %	
Depression			
Normal	77.1	46.7	0.013
Mild to moderate	17.1	33.3	
Severe to extremely severe	5.7	20.0	
Anxiety			
Normal	68.6	35.0	0.003
Mild to moderate	22.9	30.0	
Severe to extremely severe	8.6	35.0	
Stress			
Normal	88.6	58.3	0.004
Mild to moderate	11.4	21.7	
Severe to extremely severe	0	20.0	

#### Predictors of academic performance

Unadjusted analyses of academic performance with various sample characteristics showed that sleep quality and academic year were significantly associated with academic achievement. Poor sleepers were 42% less likely to have been low achievers compared to good sleepers (OR = 0.42, 95% CI: 0.17–0.99%). Similarly, preparatory-year students were 19% less likely to be low achievers. The associations remained more or less the same in the multivariate model, but lost their statistical significance. The regression model is summarised in Table 3.

**Table 3:** Association of high academic performance with sleep quality and the academic year (n = 95)

	N	Univariate		Multivariate	
		OR	95% CI	OR	95% CI
Sleep quality					
Poor sleep	60	0.42*	0.17–0.99	0.39	0.15–1.00
Good sleep	35	1.00	Ref	1.00	Ref
Academic year					
PYP	19	0.19**	0.05–0.66	0.31	0.07–1.30
Clinical year	26	0.84	0.32–2.19	0.50	0.11–2.22
Preclinical year	50	1.00	Ref	1.00	Ref

Models were additionally adjusted for the following variables: age, gender, and Quran memorization. *p-value < 0.05; **p-value < 0.01

## Discussion

The primary objective was to assess the prevalence of poor sleep quality among college students in Saudi Arabia. Results showed that almost two-thirds (63.2%) of the students were poor sleepers. These numbers were considerably higher than numbers reported by other students in the Middle East: 37.1% in Lebanon [17] and 55.7% in Egypt [18]. Studies in Saudi Arabia reported similar percentages (70–76%) [7–9].

Another objective was to assess the relationship between poor sleep quality and students’ lifestyle, academic performance, stress, anxiety, and depression levels. The results of this study showed that poor sleepers were significantly more likely to be physically inactive than good sleepers. This aligns with the results of other studies that demonstrated a bidirectional relationship between sleep and physical activity [19].

Results showed that almost 60% of the poor sleepers were high achievers. In fact, high achievers were 42% more likely to be poor sleepers when compared to low achievers. However, students who were poor sleepers were more likely to score high on the depression, anxiety, and stress scales. Other studies have reported that academic performance is associated with the timing of sleep and wakefulness, but not with total sleep duration [20].

Depression, anxiety, and stress in this sample were 42.1%, 52.6%, and 30.5%, respectively. These proportions are comparably lower than proportions reported by other Middle Eastern studies (depression, anxiety, and stress were 65%, 73%, and 59.9%, respectively) [18]. Results show that poor sleep is significantly related to the presence of depression (*p* = 0. 03), anxiety (*p *= 0.007), and stress, (*p* = 0.01). Those findings are similar to findings reported by other studies in the Middle East [18].

### Conclusion

In conclusion, this study showed that poor sleep habits were associated with a higher level of depression, anxiety, and stress among medical students. Despite this, students with poor sleep habits had higher academic performance than students with better sleep habits. Medical students should be aware of the importance of sleep quality and how it affects their mental health.

## Limitations

The current study had several limitations. The response rate of 46% of the eligible students was lower than its corresponding estimate from other studies [17, 20]. Medical students are overwhelmingly busy with their studies and have immense test anxiety, which might explain a low response rate (46%) as well as a high rate of incomplete data (50%) among those who responded. That the questions in the survey were vague or difficult is an unlikely explanation for the missing data since the majority of the records were completely empty. The length of the survey also does not explain the low response rate. Whatever the cause, this high rate of missing data seriously compromised the study, and this needs to be taken into account while interpreting the findings.

## Data Availability

The datasets generated and/or analysed during the current study are available in the Synapse repository, https://www.synapse.org/#!Synapse:syn20697344/files/.

## Abbreviations

DASS-21: Depression Anxiety Stress Scales
GPA: grade point average
PSQI: Pittsburgh Sleep Quality Index
